# The Ndc80 Loop Region Facilitates Formation of Kinetochore Attachment to the Dynamic Microtubule Plus End

**DOI:** 10.1016/j.cub.2010.12.050

**Published:** 2011-02-08

**Authors:** Jean-François Maure, Shinya Komoto, Yusuke Oku, Akihisa Mino, Sebastiano Pasqualato, Kayo Natsume, Lesley Clayton, Andrea Musacchio, Tomoyuki U. Tanaka

**Affiliations:** 1 Wellcome Trust Centre for Gene Regulation & Expression, University of Dundee, Dundee DD1 5EH, UK; 2 Department of Experimental Oncology, European Institute of Oncology, 20139 Milan, Italy

## Abstract

Proper chromosome segregation in mitosis relies on correct kinetochore-microtubule (KT-MT) interactions. The KT initially interacts with the lateral surface of a single MT (lateral attachment) extending from a spindle pole and is subsequently anchored at the plus end of the MT (end-on attachment) [1]. The conversion from lateral to end-on attachment is crucial because end-on attachment is more robust [2–4] and thought to be necessary to sustain KT-MT attachment when tension is applied across sister KTs upon their biorientation [1]. The mechanism for this conversion is still elusive. The Ndc80 complex is an essential component of the KT-MT interface [1, 5], and here we studied a role of the Ndc80 loop region, a distinct motif looping out from the coiled-coil shaft of the complex [6], in *Saccharomyces cerevisiae*. With deletions or mutations of the loop region, the lateral KT-MT attachment occurred normally; however, subsequent conversion to end-on attachment was defective, leading to failure in sister KT biorientation. The Ndc80 loop region was required for Ndc80-Dam1 interaction and KT loading of the Dam1 complex, which in turn supported KT tethering to the dynamic MT plus end [3, 7]. The Ndc80 loop region, therefore, has an important role in the conversion from lateral to end-on attachment, a crucial maturation step of KT-MT interaction.

## Graphical abstract

**Graphical Abstract:**

**Figure:**
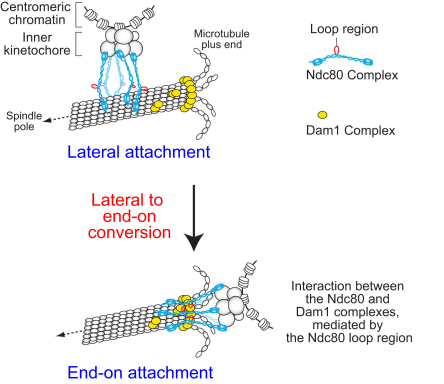

**Highlights:**

► Kinetochores must be anchored at the dynamic microtubule plus ends in early mitosis ► The Ndc80 loop region is crucial in kinetochore anchoring at the microtubule ends ► The Ndc80 loop region facilitates the interaction with the Dam1 complex ► Ndc80-Dam1 interaction ensures kinetochore anchoring at the microtubule plus ends

## Results and Discussion

**Figure 1:**
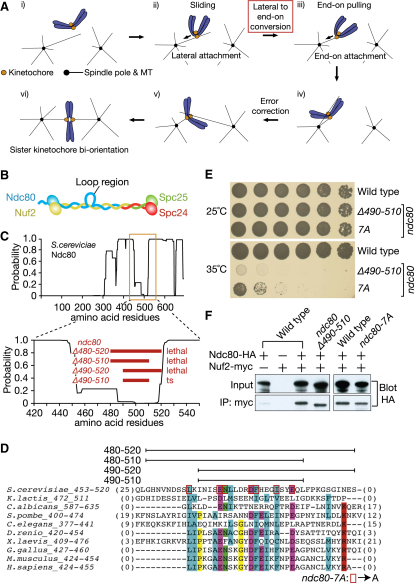
Deletions and Mutations within the Ndc80 Loop Region Cause Cell Lethality or Temperature-Sensitive Cell Growth (A) Step-wise development of kinetochore (KT)-microtubule (MT) interaction during prometaphase (i–v) and metaphase (vi). See more detail in [1]. (B) Structure of the Ndc80 complex, which consists of four proteins [8–10]. The position of the loop region is indicated. (C) The probability of forming coiled-coil motifs along amino acid residues of Ndc80 protein in *Saccharomyces cerevisiae*. Top: Full length of Ndc80. Bottom: Amino acid residues 420–550. Thick red lines indicate the positions of deletions in Ndc80 mutants, constructed in this study. ts, temperature-sensitive cell growth. (D) Multiple sequence alignment of the Ndc80 loop region from different organisms. The regions with coiled-coil probability <0.5 were selected for alignment. Some residues (numbers in parentheses) showed less conservation and were not shown here. Conserved residues are highlighted in colors: hydrophobic (light blue), acidic (purple), and basic (red) residues, asparagine (green), proline, and glycine (yellow). The positions of alanine substitution for the *ndc80-7A* mutant are shown in red rectangles. (E) *ndc80*Δ*490-510* and *ndc80-7A* mutants show temperature-sensitive cell growth. 10-fold serial dilutions of wild-type (T6500), *ndc80*Δ*490-510* (T6566), and *ndc80-7A* (T7881) cells were spotted onto YPD plates and incubated at the 25°C (top) and 35°C (bottom) for 48 hr. (F) Wild-type and *ndc80*Δ*490-510*, *ndc80-7A* cells show similar Ndc80 expression levels and similar Nuf2 association with Ndc80. *NUF2-myc* cells with *NDC80* wild-type (T7082), *ndc80*Δ*490-510* (T7085), or *ndc80-7A* (T8357), tagged with *HA*, were treated with α factor, released to fresh YPD medium at 35°C, and harvested after 70 min from the release (at which time the majority of cells were in metaphase). Wild-type cells without *HA* or *myc* tags (T7084, T6981) were also treated in the same way as controls. Total proteins (top) and the proteins immnunoprecipitated with a myc antibody (bottom) were detected by western blotting with an HA antibody.

KT-MT interaction develops in a step-wise manner [1]. The KT initially interacts with the MT lateral surface (lateral attachment) and slides along the MT towards a spindle pole (Figure 1A, i, ii). Then, the KT is tethered at the end of the MT (end-on attachment) and transported further as the MT shrinks (end-on pulling) (Figure 1A, iii). Subsequently both sister KTs interact with MTs, and aberrant KT-MT interactions are removed by error correction (Figure 1A, iv, v) until sister KT biorientation (i.e., sister KTs attaching to MTs from opposite spindle poles) is established (Figure 1A, vi).

The Ndc80 complex is an outer (i.e., closer to the MT) KT component, composed of four proteins (Figure 1B), and has a central role in comprising the KT-MT interface [1, 5]. The Ndc80 complex binds directly to the MT lateral surface in vitro, at the calponin-homology (CH) domain within Ndc80 protein (also called Hec1) [8–10], and the complex is indeed required for the lateral KT-MT attachment in vivo [11].

Presumably the Ndc80 complex is also involved in the end-on KT-MT attachment. Consistent with this, an injection of an antibody against the Ndc80 CH domain changed the dynamics of KT-MT interactions in metaphase [12]. Moreover, the Ndc80 complex can couple a microsphere at the end of a dynamic MT in an in vitro reconstituted system [13]. Thus, it is likely that the Ndc80 complex is directly involved in both the lateral and end-on KT-MT attachment. Given this, the Ndc80 complex may play a key role in the conversion from the lateral to end-on attachment.

### Mutations within the Ndc80 Loop Region Lead to Cell Lethality or Temperature-Sensitive Cell Growth

The Ndc80 complex forms a long rod-shape structure with two globular domains at each end [8–10] (Figure 1B). While one globular domain interacts with a MT, the other binds the Mtw1 complex (Mis12 complex in metazoa), a relatively inner KT component, i.e., closer to the centromere. These two globular domains are connected by long coiled-coil motifs. Peculiarly, this coiled-coil shaft is interrupted in the middle of Ndc80 protein [6] by a region of 50–60 amino acid residues that does not conform to the coiled-coil structure [10] (Figure 1C), thus presumably looping out from the coiled-coil shaft and hence called the loop region (Figure 1B). Indeed, electron microscopy revealed that the coiled-coil shaft of the Ndc80 complex showed a kink or flexible bend at the position of the loop region [14]. Intriguingly, the loop region contains several evolutionarily conserved amino acid residues (Figure 1D) and probably forms a β-sheet structure that may be involved in protein-protein interaction [14].

To address the role of the Ndc80 loop region, we constructed yeast strains whose only *ndc80* harbors a deletion of 20–40 amino acid residues within the loop region, i.e., *ndc80*Δ*480-520*, *ndc80*Δ*480-510*, *ndc80*Δ*490-520*, and *ndc80*Δ*490-510* (Figure 1C). Deletions *ndc80*Δ*480-520*, *ndc80*Δ*480-510*, and *ndc80*Δ*490-520* could not support cell viability at any temperature tried (data not shown), whereas *ndc80*Δ*490-510* cells showed growth at 25°C but not at 35°C (Figure 1E). We also constructed strains whose only *ndc80* had substitution of alanines for seven conserved amino acid residues within the loop region (and thus called *ndc80-7A*; Figure 1D). The *ndc80-7A* mutant cells showed growth at 25°C but not at 35°C (Figure 1E). Such temperature-sensitive growth of *ndc80*Δ*490-510* and *ndc80-7A* cells was not due to reduced expression of mutant Ndc80 proteins or a defect in interaction with Nuf2, another component of the Ndc80 complex (Figure 1F).

### Mutations in the Ndc80 Loop Region Support Initial KT-MT Interaction Normally but Sister KT Biorientation Is Defective

**Figure 2:**
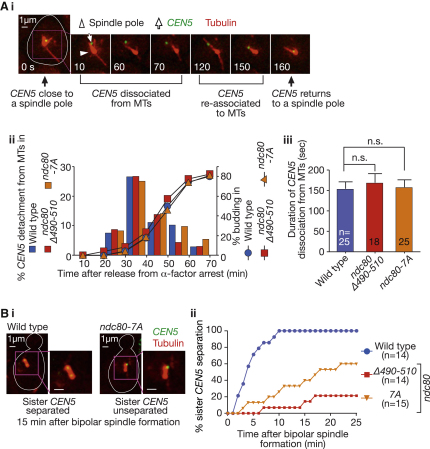
In Mutants of the Ndc80 Loop Region, the Initial KT-MT Interaction Occurs Normally, but Sister KT Biorientation Is Established Inefficiently (A) The initial KT-MT interaction: wild-type (T7848), *ndc80*Δ*490-510* (T7862), and *ndc80-7A* (T8397) cells with *CEN5-tetOs TetR-3×CFP Venus-TUB1* were treated with α factor and released to fresh YPD medium at 35°C. CFP and Venus images were acquired every 10 s at 35°C. To avoid photo-bleaching of fluorescence signals during image acquisition, the field of observation was changed every 10 min. (i) Representative live-cell images, in which a wild-type cell showed *CEN5* detachment from, and subsequent reattachment to, MTs. The cell shape is outlined in white. *ndc80*Δ*490-510* and *ndc80-7A* mutants showed similar behavior of *CEN5* (data not shown). (ii) Timing of *CEN5* detachment from MTs, shown as the percentage of cells per field showing detachment during each 10 min time window. (iii) Duration of *CEN5* dissociation from MTs in individual cells (means and standard errors). n.s., difference is not significant. (B) Establishment of sister KT biorientation. T7848, T7862, and T8397 cells (see A) were treated as in (A). CFP and Venus images were acquired every 1 min at 35°C. (i) Representative images of wild-type and *ndc80-7A* mutant cells, which showed separated and unseparated sister *CEN5*s, respectively. (ii) The percentage of cells showing separation of sister *CEN5*s on the bipolar spindle for at least two consecutive time points, until indicated time points (0 min: establishment of bipolar spindle). Sister *CEN5*s were scored as “separated” when two signals were discernible. See also Figure S2.

To address possible roles of the Ndc80 loop region in KT-MT interactions, we visualized MTs and a selected centromere (*CEN5*) by live-cell imaging and compared their behavior in wild-type, *ndc80*Δ*490-510*, and *ndc80-7A* mutant cells at 35°C. *spc24-1* mutants are defective in KT-MT attachment [11, 15] and were used as a control. In wild-type cells, *CEN5* detached from MTs (upon KT disassembly resulting from centromere DNA replication [16]) and moved away from a spindle pole. Within 2–3 min, *CEN5* interacted again with MTs when the KT was reassembled on *CEN5* [16] (Figure 2A, i). In *ndc80*Δ*490-510* and *ndc80-7A* mutant cells, *CEN5* detached from MTs and subsequently reattached to MTs with similar timing to wild-type cells (Figure 2A, ii). The duration for *CEN5* dissociation from MTs was also similar between wild-type and the loop-region mutants (Figure 2A, iii). On the other hand, *spc24-1* mutants showed earlier and longer *CEN5* dissociation from MTs, compared with wild-type (Figure S2A available online). In conclusion, mutations at the Ndc80 loop region had no effect on the efficiency of the initial interaction of KTs with MTs.

However, a subsequent step was inefficient in the Ndc80 loop-region mutants. Wild-type, *ndc80* loop-region, and *spc24-1* mutants established a bipolar spindle at the end of S phase (data not shown), and wild-type cells showed separation of sister *CEN5*s immediately afterwards (Figure 2B, i, ii), indicative of sister *CEN5* biorientation on the spindle [17, 18]. In *ndc80-7A* and *ndc80*Δ*490-510* mutant cells, sister *CEN5*s were on the spindle but their separation was delayed (Figure 2B, i, ii). In most *spc24-1* cells, sister *CEN5*s remained unseparated and did not localize on the spindle (Figure S2B). Thus, in mutants of the Ndc80 loop region, the establishment of sister KT biorientation is defective although KTs are caught on the spindle.

Meanwhile, the *ndc80-7A* and *ndc80*Δ*490-510* mutants also showed failure to satisfy the spindle-assembly checkpoint [19] (Figure S2C). We also compared the nature of the biorientation defect in these mutants with that found in *ipl1* and *mps1* mutants [1] (Figure S2D).

### The Ndc80 Loop Region Is Required for the Efficient Conversion from Lateral to End-on KT-MT Attachment

**Figure 3:**
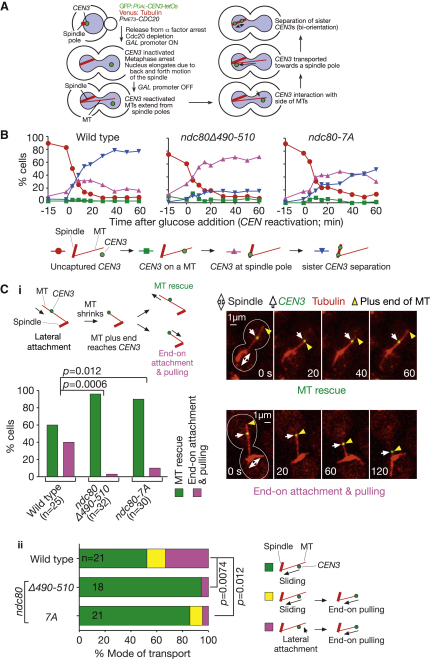
Mutants of the Ndc80 Loop Region Show Inefficient Conversion from Lateral to End-on KT-MT Attachment (A) Experimental system to analyze KT interaction with individual MTs. See details in [11]. (B) Mutants of the Ndc80 loop region show normal initial KT interaction with MTs, but subsequent establishment of sister KT biorientation is inefficient. *NDC80* wild-type (T6803), *ndc80*Δ*490-510* (T6690), and *ndc80-7A* (T7955) cells with *P_GAL_-CEN3-tetOs TetR-GFP Venus-TUB1 P_MET3_-CDC20* were treated with α factor in methionine drop-out medium with 2% raffinose for 2.5 hr and released to YP medium containing 2% galactose, 2% raffinose, and 2 mM methionine at 25°C to inactivate *CEN3* and arrest cells in metaphase. After 3 hr, the culture temperature was changed to 35°C. After 15 min, cells were suspended in the same medium but containing 2% glucose instead of galactose/raffinose to reactivate *CEN3* (defined as 0 min). Cells were collected at indicated time points and fixed with paraformaldehyde. GFP and Venus images were acquired and *CEN3*-MT interaction was scored as indicated in the schematic drawing. In most of *spc24-1* cells analyzed in this assay, *CEN3* remained uncaptured by MTs for 60 min (Figure 2c in [11]), in contrast to the *ndc80* loop-region mutants. (C) Mutants of the Ndc80 loop region show inefficient conversion from the lateral to end-on KT-MT attachment. T6803, T6690, and T7955 cells (see B) were treated as in (B), except that cells were suspended in synthetic complete medium containing 2% glucose and 2 mM methionine to reactivate *CEN3*. Cells were immobilized and GFP and Venus images were acquired every 20 s for 30 min at 35°C. (i) When the plus end of a shrinking MT caught up with *CEN3*, the MT subsequently showed either regrowth (MT rescue) or tethering of *CEN3* to its plus end while shrinking further (end-on attachment and end-on pulling). Representative images of the events in wild-type cells and a graph showing frequency of the two events; these events happened in two mutants as in wild-type cells, albeit with very different frequencies. (ii) Frequency of each mode of *CEN3* transport by a MT toward a spindle pole. Modes were classified as indicated by the schematic drawing. Sliding and end-on pulling were scored only when *CEN3* moved for 1 μm or longer by each mode of the transport. The pink bars represent the cases where the end-on attachment was established before *CEN3* moved along the MT lateral side more than 1 μm.

To analyze the KT-MT attachment of Ndc80 loop mutants in more detail, we next used an engineered assay system, in which the assembly of the KT was delayed on a particular centromere (*CEN3*) by the activation of transcription from an adjacently inserted promoter (Figure 3A) [11]. This procedure prevented *CEN3* from localizing on the mitotic spindle. While cells were arrested in metaphase, we reactivated *CEN3*, which led to KT reassembly and interaction with MTs extending from a spindle pole (spindle-pole MTs). This assay allowed observation of the individual KT-MT interaction with high spatial resolution because *CEN3* moved away from the spindle prior to its reactivation [11].

In agreement with the results in Figure 2, in *ndc80*Δ*490-510* and *ndc80-7A* mutant cells, *CEN3* was captured by the lateral surface of a spindle-pole MT at 35°C with similar kinetics as wild-type cells; by contrast, subsequent sister *CEN3* separation on the spindle proceeded more slowly compared with wild-type cells, indicative of a delay in sister *CEN3* biorientation (Figure 3B). On the other hand, in *spc24-1* cells, the initial *CEN3* capture by MTs was defective [11].

By using live-cell imaging, we investigated *CEN3*-MT interaction in further detail. In wild-type cells, after the initial *CEN3*-MT interaction, *CEN3* moved by sliding along a MT lateral surface toward a spindle pole [11]. While *CEN3* was on the MT lateral surface, this MT often underwent depolymerization at its plus end and shrank until its plus end caught up with *CEN3* (Figure 3C, i). When this happened, either of the following two events occurred in wild-type cells [3]: (1) *CEN3* was tethered at the MT end (end-on attachment) and pulled toward a spindle pole as the MT shrank further (end-on pulling) (40% of cases) or (2) the MT showed regrowth (MT rescue at *CEN3*) (60% of cases) (Figure 3C, i).

In *ndc80*Δ*490-510* and *ndc80-7A* mutant cells, *CEN3* sliding occurred almost normally, except for a small number (<5%) of *ndc80*Δ*490-510* cells showing *CEN3* pausing on a MT during an extended period (data not shown). Remarkably, in both *ndc80*Δ*490-510* and *ndc80-7A* mutants, the end-on attachment was rarely established at 35°C (Figure 3C, i), thus making subsequent end-on pulling infrequent (Figure 3C, ii), compared with wild-type cells. Thus, Ndc80 loop region is required for the efficient conversion from lateral to end-on KT-MT attachment.

Notably, defects in end-on attachment correlate well with defects in sister KT biorientation. For example, the *ndc80-7A* mutant showed milder defects in both end-on attachment and biorientation, compared with *ndc80*Δ*490-510* (see Figures 2B, 3B, and 3C). We speculate that end-on attachment might be a prerequisite for biorientation. Consistent with this, it is suggested that end-on attachment is necessary to sustain KT-MT attachment when sister KT biorientation is established and tension is applied on the KT-MT interaction [2, 4]. Indeed, the end-on attachment seems to be more robust than the lateral attachment [1, 3].

### The Ndc80 Loop Region Is Required for Ndc80-Dam1 Interaction and for Dam1 Loading on the KT

The Dam1 complex (also called DASH complex), composed of 10 proteins including Dam1 protein, also has an important role in end-on KT-MT attachment [1, 20]. In contrast to the Ndc80 complex, the Dam1 complex is not a part of the KT during the lateral KT-MT attachment and is loaded on the KT only upon end-on attachment [3].

The Dam1 complex has the ability to track the plus end of a shrinking MT [3, 7] and, once loaded on the KT, it mediates the end-on pulling of the KT by a shrinking MT [3]. During this process, the Dam1 complexes form oligomers and/or a ring structure encircling a MT [21]. Thus, the Ndc80 loop and the Dam1 complex may work together to support end-on KT-MT attachment. In this regard, it is intriguing that the Ndc80 and Dam1 complexes showed a physical interaction [22–25]. It was difficult to detect this interaction conclusively via coimmunoprecipitation or a protein pull-down (data not shown), but it could be detected with a yeast two-hybrid assay [23].

**Figure 4:**
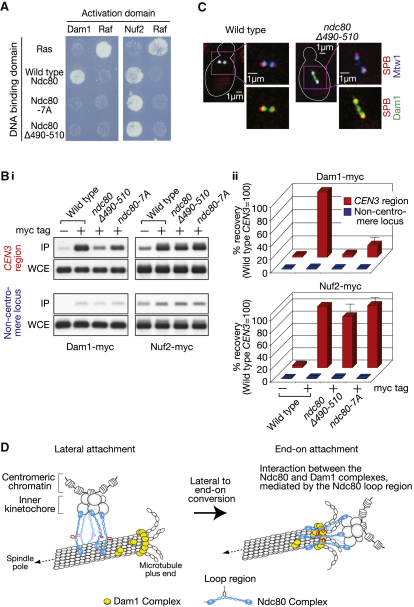
The Ndc80 Loop Region Is Required for Ndc80-Dam1 Interaction and for Dam1 Loading on the KT (A) The Ndc80 loop region is required for Ndc80-Dam1 interaction in a two-hybrid assay. The same amount of cells expressing indicated proteins, fused with a DNA binding domain or an activation domain, were spotted on histidine drop-out plates and incubated at 35°C for 48 hr. Cell growth suggests interaction between the two relevant proteins. Ras and Raf were used as controls for the assay. (B) The Ndc80 loop region is required for Dam1 loading on the KT. *DAM1-myc* cells with wild-type (T8761), *ndc80*Δ*490-510* (T8762), and *ndc80-7A* (T8763) were treated with α factor, released to fresh YPD medium at 35°C, harvested after 70 min from the release (at which time the majority of cells were in metaphase), and treated with formaldehyde to crosslink. *NUF2-myc* cells with wild-type (T8777), *ndc80*Δ*490-510* (T8778), and *ndc80-7A* (T8779) were treated in the same way. Wild-type cells without *myc* tags (T6500) were also treated in the same way, as a control. (i) Gel images of PCR products, amplified at *CEN3* region and at a noncentromere locus (*MPS1* locus, 45 kb from *CEN4*), with total DNA from whole cell extract (WCE) or immunoprecipitated DNA (IP) as a template. (ii) The percentage of recovered DNA was first quantified as a fraction of corresponding WCE in individual samples. Then, these percentage values were standardized, relative to that in *NDC80* wild-type cells (at *CEN3* region). Mean and standard errors were obtained from three independent experiments. (C) The Ndc80 loop region is required for Dam1 colocalization with the KT. Wild-type (T7868) and *ndc80*Δ*490-510* (T7866) cells with *DAM1-3×GFP MTW1-3×CFP SPC42-RFP* were cultured and harvested as in (B). Representative images are shown here. Spindle pole bodies (SPBs) were visualized with Spc42-RFP. Other representative images and the quantification of total Dam1 and Mtw1 signals in individual cells are shown in Figure S4A. (D) Summary for the role of the Ndc80 loop region (shown in red) in the conversion of lateral to end-on KT-MT attachment. During lateral attachment, the Ndc80 complex (blue) binds a MT, presumably at its Ndc80/Nuf2 CH domains and the N-terminal region of Ndc80 [8–10]. To convert lateral attachment to end-on attachment, it is crucial that the Ndc80 loop region mediates the interaction with the Dam1 complex (yellow), which localizes at the MT plus end and forms an oligomer and/or a ring encircling the MT [3, 7, 21]. The Ndc80-Dam1 interaction could be direct or indirect, and more factors might be involved in this interaction. See also Figure S4.

We therefore addressed whether the interaction between Ndc80 and Dam1 was dependent on the Ndc80 loop region by using a yeast two-hybrid assay. We first confirmed that all the wild-type Ndc80 and its mutants Ndc80Δ490-510 and Ndc80-7A showed interaction with Nuf2 (Figure 4A, right), consistent with the result in Figure 1F. We also found that wild-type Ndc80 showed a positive interaction with Dam1, as reported previously [23]. However, Ndc80Δ490-510 and Ndc80-7A mutants showed very little interaction with Dam1 (Figure 4A, left). Thus the loop region indeed facilitates interaction between Ndc80 and Dam1.

What is the functional consequence of the Ndc80-Dam1 interaction? The Ndc80 complex is required for loading of the Dam1 complex on the KT [22, 26] and an Ndc80-Dam1 interaction may facilitate this process. If so, the Ndc80 loop region might be required for Dam1 complex loading on the KT. We tested this possibility by using chromatin immunoprecipitation. In wild-type cells, centromere DNA (*CEN3*) was clearly precipitated with the Dam1 protein and also with the Nuf2 protein (Figure 4B, i, ii). Remarkably, in *ndc80*Δ*490-510* and *ndc80-7A* mutants, *CEN3* precipitation with Dam1 was considerably reduced (Figure 4B, i, ii), although *CEN3* precipitation with Nuf2 was similar between the mutants and wild-type. This result suggests a defect in Dam1 loading on KTs in these mutants.

We also compared the localization pattern of Dam1 and Mtw1 in metaphase (Figure 4C; Figure S4A). Mtw1 is a component of the KT [20] and should represent the position of KTs. Dam1 and Mtw1 showed almost perfect colocalization in wild-type cells. In *ndc80*Δ*490-510* cells, the total amount of Mtw1 and Dam1 on the spindle was not altered (Figure S4A), but Dam1 signals were often present between two globular Mtw1 signals (Figure 4C; Figure S4A). Results in Figures 4B and 4C suggest requirement of the Ndc80 loop region for Dam1 loading on the KT.

### The Ndc80 Loop Region Facilitates Interaction with the Dam1 Complex to Anchor the KT at the Dynamic MT Plus End

Our study has revealed that the Ndc80 loop region mediates the interaction with the Dam1 complex to ensure proper KT-MT attachment (Figure 4D). With Ndc80 loop-region mutants, the lateral KT-MT attachment is still largely normal; consistently, this process does not require the Dam1 complex [11]. On the other hand, the Dam1 complex has an important role in the end-on KT-MT attachment and subsequent end-on pulling of the KT by a MT [3, 7]. With Ndc80 loop region mutants, the Ndc80 and Dam1 complexes cannot interact properly, leading to the failure in the end-on attachment.

It was recently demonstrated that the Dam1 complex is able to enhance MT binding of the Ndc80 complex (e.g., its cosedimentation with MTs) in vitro [24, 25]. Given this, by using a condition reported in [24], we evaluated MT cosedimentation of the purified Ndc80 complex with loop mutants; its enhancement by the Dam1 complex was similar to that of the wild-type Ndc80 complex (Figure S4B). We reason that the loop-dependent Ndc80-Dam1 interaction in vivo was not recapitulated in this particular condition in vitro. Alternatively, an additional factor, which is missing in the in vitro reaction, may be necessary for the interaction between Dam1 and the Ndc80 loop region.

In this regard it is intriguing that, in fission yeast, Dis1 (an ortholog of Stu2 in budding yeast and XMAP215/chTOG in vertebrates) showed interaction with the Ndc80 loop region [27]. However, in contrast to fission yeast, Ndc80 and Stu2 showed no interaction in budding yeast (Figure S4C) and Ndc80 loop mutants did not alter Stu2 localization at KTs (Figure S4D). Nonetheless, Stu2 shows interaction with Dam1-complex components in a two-hybrid assay ([23]; data not shown). Thus we cannot exclude the possibility that Stu2 (possibly at the end of a shrinking MT) is involved in the Ndc80-Dam1 interaction.

Our finding that the Ndc80 loop region mediates the interaction with the Dam1 complex is consistent with nanometer-scale mapping of KT components in metaphase [28]. The Ndc80 complex bridges between the inner KT and a MT, and its Ndc80/Nuf2 globular head locates further outside (away from the inner KT) of the Dam1 complexes (see Figure 4D). In this configuration, the location of the Ndc80 loop region along the KT-MT axis approximately corresponds to that of the Dam1 complex [28].

The lateral KT-MT attachment has advantages for the initial KT-MT interaction because the MT lateral surface provides a large contact surface, whereas the end-on attachment ensures more robust KT-MT interaction [2–4], which is presumably required for sister KT biorientation. Thus the conversion from lateral to end-on attachment is an inevitable vital step in developing a proper KT-MT interaction. The Ndc80 and Dam1 complexes play central roles in comprising the KT-MT interface. Our study has identified the Ndc80 loop as an important mediator of the Ndc80-Dam1 interaction, whose role is to facilitate the crucial maturation step of the KT-MT interaction.

## Supplemental Information

Document S1. Supplemental Experimental Procedures and Two Figures
