# Supplementary material for: The Ndc80 Loop Region Facilitates Formation of Kinetochore Attachment to the Dynamic Microtubule Plus End
Source: Curr Biol. 2011 Feb 8;21(3):207–13. doi: 10.1016/j.cub.2010.12.050 (PMC3052438; doi:10.1016/j.cub.2010.12.050)
Supplement: Document S1. Supplemental Experimental Procedures and Two Figures [file mmc1.pdf]

Current Biology, Volume *21*

Supplemental Information

The Ndc80 Loop Region Facilitates Formation of Kinetochore Attachment to the Dynamic Microtubule Plus End

Jean-François Maure, Shinya Komoto, Yusuke Oku, Akihisa Mino, Sebastiano Pasqualato, Kayo Natsume, Lesley Clayton, Andrea Musacchio, and Tomoyuki U. Tanaka

Supplemental Information

Inventory

pages 2-4. Supplemental Figure S2 (associated with Figure 2) and its legend

pages 5-8. Supplemental Figure S4 (associated with Figure 4) and its legend

pages 9-13. Experimental procedures

pages 14-16. References cited in supplemental information

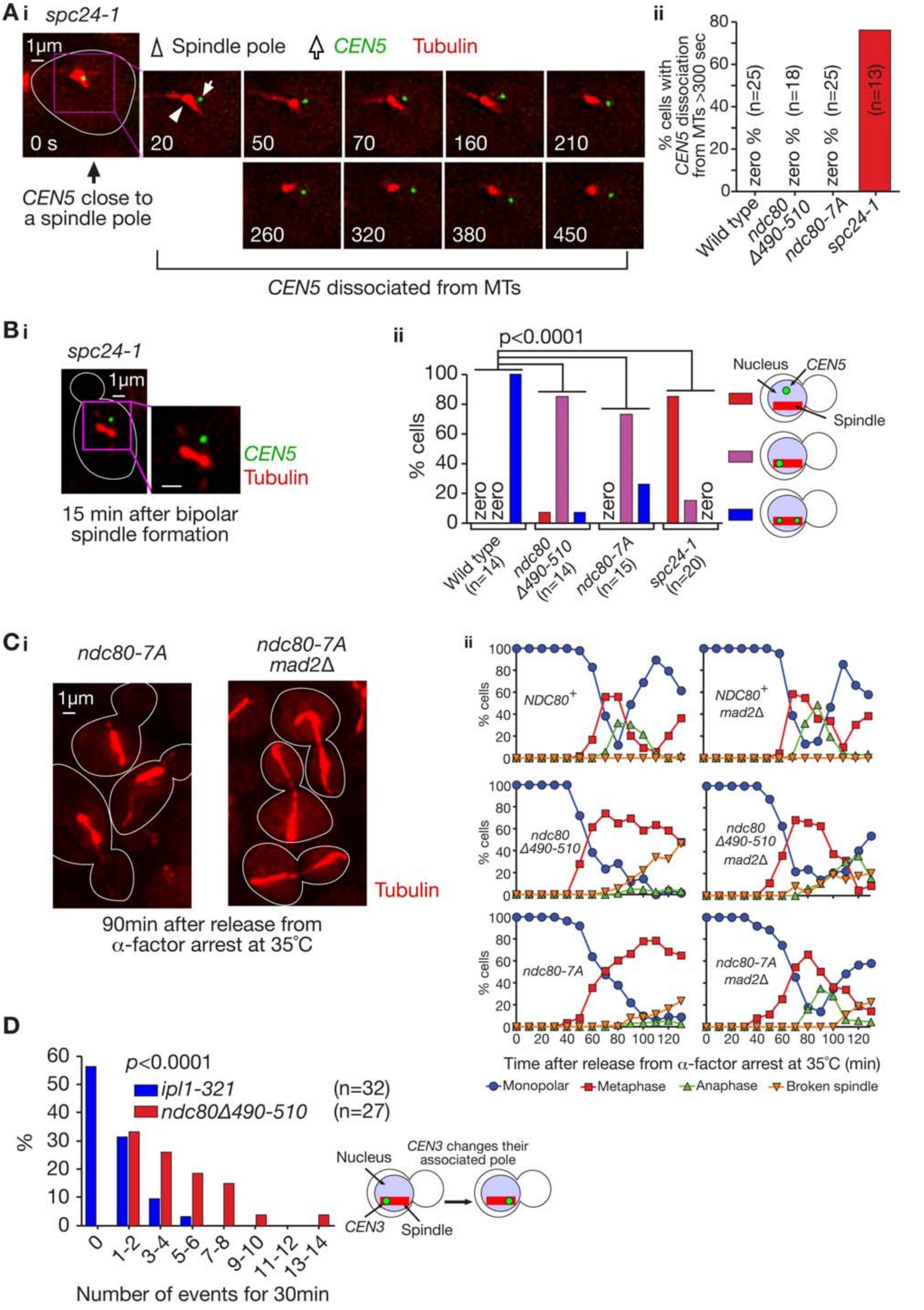

Figure S2 (Supplemental figure associated with Figure 2)

Figure S2 (associated with Figure 2).

A. *spc24-1* cells show a delay in the initial KT-MT interaction (a control associated with Fig 2A).

spc24-1 cells (T9168) with *CEN5-tetOs TetR-3×CFP Venus-TUB1* were treated as in Fig 2A. (i) A representative live-cell image, in which an *spc24-1* cell showed *CEN5* detachment from MTs without subsequent re-association with MTs. The cell shape is outlined in white. (ii) The percentage of cells in which *CEN5* did not show re-association with MTs for 300 sec or longer, following observed detachment from MTs (cells with *CEN5* dissociation at the beginning of a 10-min time window of observation were not included in the results here and Fig 2A ii, iii). Note that, at 20 min after release from α factor, 21/57 (37%) of *spc24-1* cells already showed *CEN5* dissociation from MTs. In the same timing, none of wild-type, *ndc80Δ490-510* or *ndc80-7A* cells (0/66, 0/69 and 0/72, respectively) showed *CEN5* dissociation from MTs.

B. In *spc24-1* cells, sister *CEN5*s remain unseparated and do not localize on the bipolar spindle (a control associated with Fig 2B).

T9168 cells (see A) were treated as in Fig 2B. (i) A representative image of an *spc24-1* cell, in which sister *CEN5*s remained unseparated and did not localize on the bipolar spindle at 15 min after establishment of the bipolar spindle. (ii) The percentage of cells showing unseparation of sister *CEN5*s that were not on the bipolar spindle, separated (at least for two consecutive time points)/unseparated sister *CEN5*s that were on the spindle, until 15 min after establishment of bipolar spindle.

C. Ndc80 loop mutants fail to satisfy the spindle-assembly checkpoint.

Wild-type (T7848) and *ndc80Δ490-510* (T7862) and *ndc80-7A* (T8397) cells with *CEN5-tetOs TetR-3×CFP Venus-TUB1 SCC1-4×mCherry* $MAD2^{+}$ were treated with α factor and released to fresh YPD medium at 35 °C (at 0 min). Cells with the same genotypes but with deletion of *mad2* (T7849, T7861 and T8157, respectively) were also treated in the same way. Cell aliquots were collected every 10 min and fixed with paraformaldehyde, followed by acquisition of CFP, Venus and mCherry images. (i) Representative images of *ndc80-7A* and *ndc80-7A mad2*Δ cells at 90 min. (ii) Spindles in individual cells were classified as follows, based on their morphologies and the presence/absence of Scc1 signals in the nucleus: Monopolar, spindle with a single pole; Metaphase, bipolar spindle with length < 4 µm and positive Scc1 signals in the nucleus; Anaphase, bipolar spindle with length > 4 µm and very little Scc1 signal in the nucleus: Broken spindle, broken spindle or long bipolar spindle > 4 µm with positive Scc1 signals in the nucleus.

Results: the defect in sister KT bi-orientation led to failure to satisfy the spindle-assembly checkpoint (SAC) in *ndc80Δ490-510* and *ndc80-7A* mutants of the Ndc80 loop region. These mutants showed a delay in anaphase onset, which was dependent on *MAD2*, a key component of SAC [1]. A defect in sister KT bi-orientation was also found in *ipl1* (Ipl1 is an orthologue of metazoan Aurora B) and *mps1* mutants; however, in contrast to the *ndc80* loop region mutants, they did not engage the SAC [2-4]. We also found that, in *ndc80Δ490-510* and *ndc80-7A* cells with *mad2* deletion, sister *CEN5*s showed frequent missegregation during anaphase (56 % at 110 min and

48 % at 90 min, respectively), in contrast to wild-type *NDC80* with *mad2* deletion (0 %).

D. Mono-oriented sister *CEN5*s often change their associated spindle poles in the Ndc80 loop mutants, in contrast to *ipl1* mutants.

ndc80Δ490-510 (T6690) and *ipl1-321* (T2863) cells with *P_{GAL}-CEN3-tetOs TetR-GFP Venus(or YFP)-TUB1 P_{MET3}-CDC20* were treated with α factor in methionine drop-out medium with 2 % glucose and released to YP medium containing 2 % glucose and 2 mM methionine at 35 °C to arrest cells in metaphase. After 2.5 hrs, cells were immobilized and GFP and Venus (YFP) images were acquired every 20 sec for 30 min. The number of events, in which mono-oriented sister *CEN3*s changed their associated spindle poles on the spindle, were counted and shown in the graph.

Results: a defect in sister KT bi-orientation was also found in mutants of Ipl1 and Mps1, which facilitate turnover of KT-MT attachment with aberrant orientation [3, 5]. We compared behaviour of mono-oriented *CEN5* in the Ndc80 loop mutants, *ipl1* and *mps1* mutants. When sister *CEN5*s failed to bi-orient in both the Ndc80 loop region and *ipl1* mutants, they remained unseparated and located in the vicinity of a spindle pole and this was also the case for *mps1* mutants [5]. Intriguingly, whereas the associated sister *CEN5*s often changed their associated spindle poles in the *ndc80* loop region mutants (red bars), such changes were infrequent in *ipl1* and *mps1* mutants (blue bars for *ipl1*) [3, 5]. Thus, Ndc80 loop region mutants may still be able to facilitate turnover of KT-MT attachment, in contrast to *ipl1* and *mps1* mutants. The results in Figs S2C and S2D are consistent with the possibility that the Ndc80 loop region facilitates sister KT bi-orientation with a different mechanism, at least in part, from that of Ipl1 and Mps1 kinases.

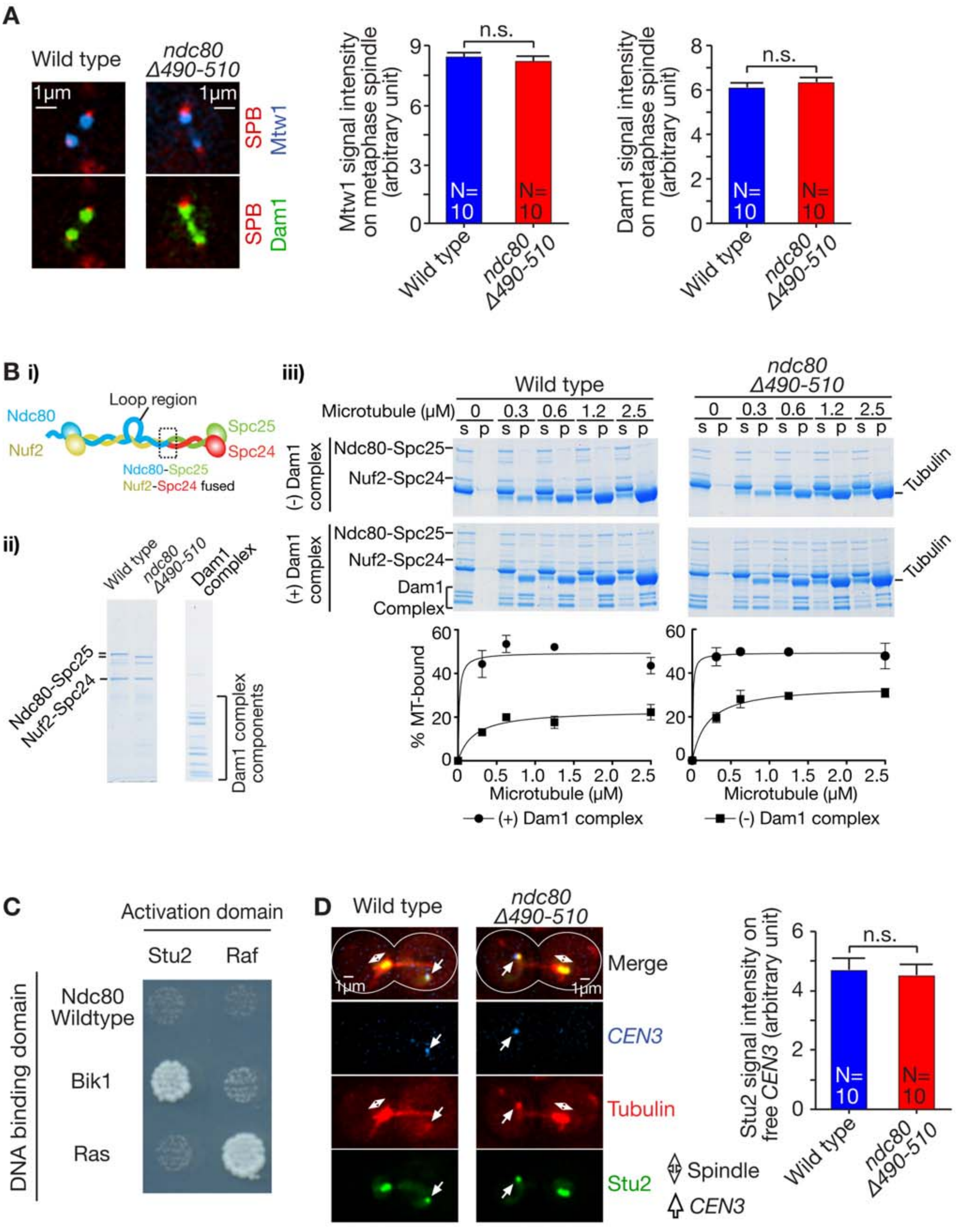

Figure S4 (Supplemental figure associated with Figure 4)

Figure S4 (associated with Figure 4)

A. The Ndc80 loop region is required for Dam1 co-localization with the KT.
Wild-type (T7868) and *ndc80Δ490-510* (T7866) cells with *DAM1-3×GFP MTW1-3×CFP SPC42-RFP* were cultured and harvested as in Fig 4C, and observed with microscopy. A representative image of each cell and quantification (means and standard errors) of total Dam1 and Mtw1 signals in individual cells are shown here. SPB: spindle pole body, visualized with Spc42-RFP. *n.s.*: difference is not significant. Another representative image is shown in Fig 4C.

Results: In most (14/15) of wild-type cells, Dam1 signals (> 90% in projected images) co-localized almost perfectly with Mtw1 signals. On the other hand, in most (13/16) of *ndc80Δ490-510* cells, a fraction of Dam1 signals (20-50 %) did not show co-localization with Mtw1 signals. Thus the Ndc80 loop region is required for Dam1 co-localization with KTs. In addition, we quantified sums of Dam1 and Mtw1 signals between two SPBs in individual cells. In this quantification, wild-type and *ndc80Δ490-510* cells did not show significant difference. Thus the Ndc80 loop region mutant disrupts co-localization between Dam1 and Mtw1 (Mtw1 is representing the KT positions) without changing their total amounts on the metaphase spindle.

B. In a MT co-sedimentation assay *in vitro*, the Dam1 complex enhances MT binding of the Ndc80 complexes similarly between *NDC80* wild-type and *ndc80Δ490-510.*
(i) To obtain the *S. cerevisiae* Ndc80 complex from recombinant proteins in high yield, Ndc80-Spc25 and Nuf2-Spc24 fusion proteins were expressed in *E. coli*. (ii) Ndc80-Spc25 and Nuf2-Spc24 were co-purified from the bacteria. Two versions of Ndc80-Spc25 were used; one with wild-type Ndc80 and the other with *ndc80Δ490-510*. Meanwhile, 10 components of the *S. cerevisiae* Dam1 complex were also expressed in *E. coli* and co-purified. These proteins were applied and separated on SDS-PAGE gels. (iii) Microtubule co-sedimentation of the Ndc80 complex (with wild-type Ndc80 and the *ndc80Δ490-510* mutant) in the presence and absence of the Dam1 complex in a buffer containing 100 mM NaCl. Representative images of gels are shown here. Co-sedimentation of the Ndc80 complex with MTs was quantified and plotted (means and standard errors), using the data obtained from three independent experiments.

Results: Using a MT co-sedimentation assay, it was recently demonstrated that the Dam1 complex is able to enhance MT binding of the Ndc80 complex in a buffer containing 100 mM NaCl [6]. Using the same condition, we evaluated MT co-sedimentation of the purified Ndc80 complex with the loop mutant *ndc80Δ490-510*; its enhancement by the Dam1 complex was similar to that of the wild-type Ndc80 complex. Note that, for MT-binding reactions in this experiment, samples were incubated at room temperature for 20 min. Similar results were obtained when they were incubated at 35 ˚C for 20 min (data not shown). These results are consistent with the result that the isolated Ndc80-Nuf2 head was sufficient for enhancement of MT association by the Dam1 complex, under these conditions [6].

C. Stu2 and Ndc80 do not show interaction in a two-hybrid assay.
A two-hybrid assay was carried out as in Fig 4A. Bik1 was included in this assay as a control to confirm that our Stu2 construct fused with the activation domain produces a

functional fusion protein [7, 8]. We also tried to detect possible Ndc80-Stu2 interaction using co-immunoprecipitation but could not detect it (data not shown).

D. Stu2 shows localization at *CEN3* (which is reactivated, but not yet interacted with MTs or the spindle) and on the metaphase spindle, similarly between wild-type and *ndc80Δ490-510* cells.

Ndc80 wild-type (T7305) and *ndc80Δ490-510* (T7269) cells with P_{GAL}-*CEN3-tetOs TetR-3×CFP STU2-4×mCherry Venus-TUB1* P_{MET3}-*CDC20* were treated as in Fig 3B. Images were acquired 5-10 min after cells were suspended in medium with glucose (*CEN3* reactivation). Representative images and the quantification (means and standard errors) of Stu2 signals at *CEN3* are shown here. *n.s.*: difference is not significant.

Results: Using the assay shown in Fig 3A, we previously found that Stu2 localized at *CEN* after its reactivation and before its interaction with a MT extending from a spindle pole [9, 10]. This was also the case during lateral KT-MT attachment, albeit with a reduced amount of Stu2 at *CEN* [10]. The intensity of Stu2 signals at *CEN* in these conditions was similar between Ndc80 wild-type and *ndc80Δ490-510* (this figure and data not shown). The intensity of Stu2 signals on the metaphase spindle was also similar between the two strains (this figure, quantification not shown). Note that tubulin signals are associated with uncaptured *CEN3* and this is dependent on Stu2 [10]; the intensity of the tubulin signals was similar between Ndc80 wild-type and *ndc80Δ490-510* (this figure, quantification not shown).

Supplemental notes (associated with Figure 4):

1) If proper Ndc80-Dam1 interaction is necessary for correct KT-MT attachment, *dam1* mutants may also show defects in KT-MT attachment similarly to *ndc80* loop region mutants. We previously found that, in *dam1-1* mutant cells, lateral attachment happened normally, but then *CEN* was often tethered to the end of a MT that could not subsequently depolymerize; i.e. end-on pulling was defective [11]. Thus the phenotypes of the *dam1-1* and *ndc80* loop region mutants were similar, except that *CEN* was tethered at the MT end in *dam1-1* while a MT was rescued in *ndc80* loop mutants. We speculate that, in *dam1-1*, residual Dam1 function may still allow interaction with Ndc80; however the resultant end-on attachment does not lead to end-on pulling due to a defect in *dam1*.

2) It is intriguing that, when Ndc80 loop region mutants failed to establish end-on attachment, MT rescue happened rather than KT detachment from the MT. Ndc80 loop region mutants show KT-MT interaction during this process, presumably because their intact CH domain and N-terminal region can still maintain MT association [12-14]. However, we reason that such attachment would not be able to complete end-on configuration in the absence of proper Ndc80-Dam1 interaction, and MT rescue occurs, facilitated by Stu2 associated with the KT ([9]; our unpublished result).

3) Is the role of the Ndc80 loop region in the end-on KT-MT attachment conserved in evolution? The Dam1 complex is found in various yeast species, but its orthologues have not been identified in metazoa [15]. Nonetheless, several amino acid residues are

well conserved within the Ndc80 loop region between yeasts and metazoa [16] (see Fig 1D). This raises an interesting possibility that the Ndc80 loop region may facilitate interaction with functional counterparts of the Dam1 complex to establish end-on KT-MT attachment in metazoan cells. For example, in these organisms, the Ska1 complex is proposed to be a functional equivalent to the Dam1 complex [17, 18].

Experimental procedures

Yeast strains and culture

The background of yeast strains (W303) and methods for yeast culture and α factor treatment were as described previously [11]. Unless otherwise stated, cells were cultured at 25 ºC in YP medium containing glucose (YPD). *NUF2*, *DAM1*, *MTW1* and *STU2* genes were tagged at their C-termini at their original gene loci by a one-step PCR method, using 9× *myc*, 18× *myc* (C3536 and C3537, respectively, from K. Nasmyth lab), 3×*GFP* (pSM1023 from E. Schiebel lab), 3×*CFP* (pT769) or 4×*mCherry* (pT909) cassettes as PCR templates [19, 20]. *pT909* was constructed by multiplying the *mCherry* gene in pKS391 [21]. Constructs of *CEN5-tetOs* [22], P_{GAL}-*CEN3-tetOs* [9], *TetR-3×CFP* [23], P_{MET3}-*CDC20* [24], *TetR-GFP* [25], *SPC42-RFP* [20], *ipl1-321* [26] and *spc24-1* [27] were described previously. For a control in Figs 2 and 3, we initially planned to use *ndc80-1*, however it has one mutation within the loop in addition to several other mutations [27]. Instead we have chosen *spc24-1* as a control. *Venus-TUB1* was constructed through mutagenesis of *YFP-TUB1* (pDH20, obtained from Yeast Resource Centre, Seattle, USA) and was integrated at an auxotroph marker locus.

Constructing *ndc80* mutants

Mutations and deletions were introduced by site-directed mutagenesis on the *NDC80* gene using the Quick Change kit (Stratagene) following manufacturer's protocol, and verified by DNA sequencing. *NDC80* wild-type (as a control) and mutants with its original promoter and terminator were cloned into pRS303. A single copy of this plasmid was integrated at *his3* locus in a diploid strain where one of the original *NDC80* genes had been deleted at its original locus. Through tetrad dissection of spores from this diploid strain, we obtained a haploid cell whose only *NDC80* is wild-type or a mutant inserted at *his3* locus. To make a version of Ndc80 tagged with HA, six copies of tandem *HA* tags were inserted at the C-terminus of *NDC80* wild-type and mutants, which had been cloned in pRS303, and their single copy was subsequently integrated at *his3* locus similarly to a non-tagged version.

Computer program and statistical analyses

The coiled-coil probability was calculated using COILS [28]. Multiple sequences of amino acid residues were aligned using Jalview [29]. Statistical analyses were carried out using Prism (Graph Pad) software, by choosing the unpaired *t*-test (Figs 2A, S2D, S4A, S4D), Fisher's exact test for contingency table (Fig 3C i) and the chi-square test for trend (Figs S2B, 3C ii).

Live-cell imaging

The procedures for time-lapse fluorescence microscopy were described previously [30]. Time-lapse images were collected at 35 ºC unless otherwise stated. For image acquisition, we used a DeltaVision RT microscope (Applied Precision), an UPlanSApo 100× objective lens (Olympus; NA 1.40), SoftWoRx software (Applied Precision), and either a CoolSnap HQ (Photometrics) or Cascade II 512B (Roper Scientific) CCD camera. We acquired 7-9 (0.5-0.7 mm apart) z-sections, which were subsequently deconvoluted, projected to two-dimensional images and analyzed with SoftWoRx and Volocity (Improvision) software. CFP, Venus (or GFP), and mCherry (or RFP) signals were discriminated using the 89006 multi-band filter set (Chroma). GFP and Venus signals were discriminated using the JP3 filter set (Chroma). Images of fixed cells were acquired similarly except that cells had been treated with paraformaldehyde for fixation beforehand.

Protein immunoprecipitation

Protein extracts were prepared from cells by 2-min bead beating at 4˚C in buffer containing 50mM HEPES pH 7.5, 150mM NaCl, 0.05% Tween 20, 1mM β-mercaptoethanol, protease inhibitor cocktail IV (Calbiochem) and PhosSTOP phosphatase inhibitor cocktail (Roche). Extracts were cleared by centrifugation at 10,000 g for 10 min at 4˚C and equal amounts (500 μg) of total protein were used for immunoprecipitations. Myc-tagged proteins were immunoprecipitated using anti-myc agarose beads (Sigma). Beads were rolled with extracts at 4˚C for 1 hour, washed 3 times by re-suspension in the above buffer containing 0.5 M NaCl with a final wash in extraction buffer. Proteins were eluted by heating at 100˚C in Laemmli sample buffer for 3 min, separated by PAGE on 4-12% Novex Bis-Tris gels and HA-tagged proteins were detected on Western blots using 12CA5 anti-HA antibody.

Two-hybrid assay

Two-hybrid assay was carried out as described previously [31]. The constructs of *NDC80* (wild-type and mutants) and *BIK1* were amplified by PCR, cloned into *pBTM116* plasmid [32] and fused with the DNA binding domain. The constructs of *DAM1*, *NUF2* and *STU2* were amplified by PCR, cloned into *pGAD-C2* plasmid [33] and fused with the activation domain for transcription. These plasmids were transformed into the L40 strain of *S. cerevisiae*. Two-hybrid interaction was evaluated by expression of *HIS3* reporter gene on medium lacking histidine. Constructs with *RAS* and *RAF* were used as controls.

Chromatin immunoprecipitation

Chromatin immunoprecipitation was carried out as described previously [34]. The percentage of DNA recovery in immunoprecipitation was quantified by making a serial dilution of immunoprecipitated DNA and total DNA from whole cell extracts, followed by their use as PCR templates, as described previously [34]. The PCR amplified a 300-bp DNA fragment spanning *CEN3* and, as an example of a non-centromere locus, a 230-bp DNA fragment within *MPS1* gene (45 kb from *CEN4*).

Purification of recombinant protein

To obtain the purified *S. cerevisiae* Ndc80 complex in high yield, we made two fusion proteins; i.e. Ndc80 (1-584)-Spc25 (57-222) and Nuf2 (1-364)-Spc24 (84-213); numbers in parentheses show amino acid residues. The Ndc80 complex, made of two fusion polypeptides, was named the Ndc80 *sequoia* complex, as it contained the majority of Ndc80, Nuf2, Spc24 and Spc25 except for their junctions connecting Ndc80/Nuf2 and Spc24/25 heterodimers (compare with *bonsai* complex; [14]). Ndc80 *sequoia* complex was expressed in *E. coli* strain BL21(DE3)plysS (Novagen) under the control of *tac* promoter. 6 L of bacterial culture was grown at 37 ˚C to OD 0.5 and protein expression was induced by incubation with 0.3 mM IPTG at 25 ˚C for 16 hr. Cells were harvested by centrifugation, washed with 0.9 % NaCl and re-suspended in 50mM Tris-HCl (pH7.5), 0.1 % TritonX-100, 300 mM NaCl, 1 mM EDTA, 1 mM DTT, 10 % glycerol containing Complete EDTA-free protease inhibitor (Roche). Cells were disrupted by sonication, the extract was cleared by centrifugation and the lysate was incubated with glutathione-sepharose (GE Healthcare) for 2 hr at 4 ˚C. Beads were washed 5 times with lysis buffer, and GST was removed from Nuf2-

Spc24 by cleavage with Prescision protease (GE Healthcare) for 16 hr at 4˚C. Ndc80-Spc25 and Nuf2-Spc24 were purified together from the glutathione-sepharose unbound fraction and formation of a complex was confirmed by gel filtration on a Superose 6 column.

The 10 components of the *S. cerevisiae* Dam1 complex were co-expressed in *E. coli* strain Rosetta(DE3)plysS (Novagen) using pDASH-Spc34His plasmid (a gift from S. Harrison lab) as described previously [35, 36]. Protein expression was induced in 4 L of bacterial culture by the addition of 0.1 mM IPTG followed by incubation at 37 ˚C for 4 hr. Bacterial cells were harvested, washed with PBS, re-suspended with 20 mM sodium phosphate (pH6.8), 0.5 % TritonX-100, 500 mM NaCl, 1 mM EDTA, 20 mM imidazole containing Complete EDTA-free protease inhibitor (Roche) and disrupted by sonication. Extract was cleared by centrifugation and the resulting lysate was incubated with Ni-NTA (Qiagen) for 2 hr at 4˚C. Beads were collected by centrifugation, washed 5 times with the same buffer without TritonX-100 and proteins were eluted with 20 mM sodium phosphate (pH 6.8), 150 mM NaCl, 1 mM EDTA, 200 mM imidazole. Buffer was exchanged to 20 mM sodium phosphate (pH 6.8), 150 mM NaCl, 1 mM EDTA using a PD10 desalting column (GE Healthcare) and the desalted fraction was loaded on 1 ml Q-Sepharose and protein was eluted with a stepwise gradient of NaCl. Fractions were dialyzed against 20 mM sodium phosphate (pH 6.8), 150 mM NaCl, 1 mM EDTA.

Microtubule co-sedimentation assays

Assays were conducted using using tubulin polymerized with taxol (both from Cytoskeleton, Inc) in BRB80 (80mM PIPES-NaOH pH6.8, 1 mM $MgCl_2$, 1mM EGTA) containing 1mM GTP and 20 μM taxol, as described in [6, 37]. Ndc80 *sequoia* complex and Dam1 complex were cleared by centrifugation and 0.3 μM *sequoia*, in the presence or absence of 0.3 μM Dam1 complex, was mixed with microtubules in BRB80 containing 1 μM BSA and 100 mM NaCl and incubated at room temperature for 20 min, after which the reaction was centrifuged at 90,000 rpm for 10 min at 25 ˚C in TLA100 rotor. Equivalent volumes of the supernatant and pellet were run on 4-12 % Novex Bis-Tris SDS-PAGE gels (Invitrogen), which were stained with Simply Blue Safe Stain (Invitrogen) and scanned to quantify the amounts

of Ndc80-Spc25 fusion protein using Image J. Quantified data were plotted and fitted to the equation of $Y = B_{max} \times X / (K_D + X)$ to draw regression curves, using Prism 5 (GraphPad Software, Inc).

References cited in supplemental information

1. Musacchio, A., and Salmon, E.D. (2007). The spindle-assembly checkpoint in space and time. Nat Rev Mol Cell Biol *8*, 379-393.
2. Biggins, S., and Murray, A.W. (2001). The budding yeast protein kinase Ipl1/Aurora allows the absence of tension to activate the spindle checkpoint. Genes Dev *15*, 3118-3129.
3. Tanaka, T.U., Rachidi, N., Janke, C., Pereira, G., Galova, M., Schiebel, E., Stark, M.J., and Nasmyth, K. (2002). Evidence that the Ipl1-Sli15 (Aurora kinase-INCENP) complex promotes chromosome bi-orientation by altering kinetochore-spindle pole connections. Cell *108*, 317-329.
4. Winey, M., and Huneycutt, B.J. (2002). Centrosomes and checkpoints: the MPS1 family of kinases. Oncogene *21*, 6161-6169.
5. Maure, J.F., Kitamura, E., and Tanaka, T.U. (2007). Mps1 kinase promotes sister-kinetochore bi-orientation by a tension-dependent mechanism. Curr Biol *17*, 2175-2182.
6. Lampert, F., Hornung, P., and Westermann, S. (2010). The Dam1 complex confers microtubule plus end-tracking activity to the Ndc80 kinetochore complex. J Cell Biol *189*, 641-649.
7. Ito, T., Chiba, T., Ozawa, R., Yoshida, M., Hattori, M., and Sakaki, Y. (2001). A comprehensive two-hybrid analysis to explore the yeast protein interactome. Proc Natl Acad Sci U S A *98*, 4569-4574.
8. Wolyniak, M.J., Blake-Hodek, K., Kosco, K., Hwang, E., You, L., and Huffaker, T.C. (2006). The regulation of microtubule dynamics in Saccharomyces cerevisiae by three interacting plus-end tracking proteins. Mol Biol Cell *17*, 2789-2798.
9. Tanaka, K., Mukae, N., Dewar, H., van Breugel, M., James, E.K., Prescott, A.R., Antony, C., and Tanaka, T.U. (2005). Molecular mechanisms of kinetochore capture by spindle microtubules. Nature *434*, 987-994.
10. Kitamura, E., Tanaka, K., Komoto, S., Kitamura, Y., Antony, C., and Tanaka, T.U. (2010). Kinetochores generate microtubules with distal plus ends: their roles and limited lifetime in mitosis. Dev Cell *18*, 248-259.
11. Tanaka, K., Kitamura, E., Kitamura, Y., and Tanaka, T.U. (2007). Molecular mechanisms of microtubule-dependent kinetochore transport toward spindle poles. J Cell Biol *178*, 269-281.
12. Cheeseman, I.M., Chappie, J.S., Wilson-Kubalek, E.M., and Desai, A. (2006). The conserved KMN network constitutes the core microtubule-binding site of the kinetochore. Cell *127*, 983-997.
13. Wei, R.R., Al-Bassam, J., and Harrison, S.C. (2007). The Ndc80/HEC1 complex is a contact point for kinetochore-microtubule attachment. Nat Struct Mol Biol *14*, 54-59.
14. Ciferri, C., Pasqualato, S., Screpanti, E., Varetti, G., Santaguida, S., Dos Reis, G., Maiolica, A., Polka, J., De Luca, J.G., De Wulf, P., et al. (2008). Implications for kinetochore-microtubule attachment from the structure of an engineered Ndc80 complex. Cell *133*, 427-439.
15. Meraldi, P., McAinsh, A.D., Rheinbay, E., and Sorger, P.K. (2006). Phylogenetic and structural analysis of centromeric DNA and kinetochore proteins. Genome Biol *7*, R23.

16. Wang, H.W., Long, S., Ciferri, C., Westermann, S., Drubin, D., Barnes, G., and Nogales, E. (2008). Architecture and flexibility of the yeast Ndc80 kinetochore complex. J Mol Biol *383*, 894-903.
17. Hanisch, A., Sillje, H.H., and Nigg, E.A. (2006). Timely anaphase onset requires a novel spindle and kinetochore complex comprising Ska1 and Ska2. Embo J *25*, 5504-5515.
18. Welburn, J.P., Grishchuk, E.L., Backer, C.B., Wilson-Kubalek, E.M., Yates, J.R., 3rd, and Cheeseman, I.M. (2009). The human kinetochore Ska1 complex facilitates microtubule depolymerization-coupled motility. Dev Cell *16*, 374-385.
19. Knop, M., Siegers, K., Pereira, G., Zachariae, W., Winsor, B., Nasmyth, K., and Schiebel, E. (1999). Epitope tagging of yeast genes using a PCR-based strategy: more tags and improved practical routines. Yeast *15*, 963-972.
20. Maekawa, H., Usui, T., Knop, M., and Schiebel, E. (2003). Yeast Cdk1 translocates to the plus end of cytoplasmic microtubules to regulate bud cortex interactions. Embo J *22*, 438-449.
21. Snaith, H.A., Samejima, I., and Sawin, K.E. (2005). Multistep and multimode cortical anchoring of tea1p at cell tips in fission yeast. EMBO J *24*, 3690-3699.
22. Tanaka, T., Fuchs, J., Loidl, J., and Nasmyth, K. (2000). Cohesin ensures bipolar attachment of microtubules to sister centromeres and resists their precocious separation. Nat Cell Biol *2*, 492-499.
23. Bressan, D.A., Vazquez, J., and Haber, J.E. (2004). Mating type-dependent constraints on the mobility of the left arm of yeast chromosome III. J Cell Biol *164*, 361-371.
24. Uhlmann, F., Wernic, D., Poupart, M.A., Koonin, E.V., and Nasmyth, K. (2000). Cleavage of cohesin by the CD clan protease separin triggers anaphase in yeast. Cell *103*, 375-386.
25. Michaelis, C., Ciosk, R., and Nasmyth, K. (1997). Cohesins: chromosomal proteins that prevent premature separation of sister chromatids. Cell *91*, 35-45.
26. Biggins, S., Severin, F.F., Bhalla, N., Sassoon, I., Hyman, A.A., and Murray, A.W. (1999). The conserved protein kinase Ipl1 regulates microtubule binding to kinetochores in budding yeast. Genes Dev *13*, 532-544.
27. Wigge, P.A., and Kilmartin, J.V. (2001). The Ndc80p complex from Saccharomyces cerevisiae contains conserved centromere components and has a function in chromosome segregation. J Cell Biol *152*, 349-360.
28. Lupas, A., Van Dyke, M., and Stock, J. (1991). Predicting coiled coils from protein sequences. Science *252*, 1162-1164.
29. Waterhouse, A.M., Procter, J.B., Martin, D.M., Clamp, M., and Barton, G.J. (2009). Jalview Version 2--a multiple sequence alignment editor and analysis workbench. Bioinformatics *25*, 1189-1191.
30. Tanaka, K., Kitamura, E., and Tanaka, T.U. (2010). Live-cell analysis of kinetochore-microtubule interaction in budding yeast. Methods *51*, 206-213.
31. Yamagishi, Y., Sakuno, T., Shimura, M., and Watanabe, Y. (2008). Heterochromatin links to centromeric protection by recruiting shugoshin. Nature *455*, 251-255.
32. Vojtek, A.B., Hollenberg, S.M., and Cooper, J.A. (1993). Mammalian Ras interacts directly with the serine/threonine kinase Raf. Cell *74*, 205-214.

33. James, P., Halladay, J., and Craig, E.A. (1996). Genomic libraries and a host strain designed for highly efficient two-hybrid selection in yeast. Genetics *144*, 1425-1436.
34. Tanaka, T., Cosma, M.P., Wirth, K., and Nasmyth, K. (1999). Identification of cohesin association sites at centromeres and along chromosome arms. Cell *98*, 847-858.
35. Miranda, J.J., De Wulf, P., Sorger, P.K., and Harrison, S.C. (2005). The yeast DASH complex forms closed rings on microtubules. Nat Struct Mol Biol *12*, 138-143.
36. Westermann, S., Avila-Sakar, A., Wang, H.W., Niederstrasser, H., Wong, J., Drubin, D.G., Nogales, E., and Barnes, G. (2005). Formation of a Dynamic Kinetochore- Microtubule Interface through Assembly of the Dam1 Ring Complex. Mol Cell *17*, 277-290.
37. Cheeseman, I.M., Brew, C., Wolyniak, M., Desai, A., Anderson, S., Muster, N., Yates, J.R., Huffaker, T.C., Drubin, D.G., and Barnes, G. (2001). Implication of a novel multiprotein Dam1p complex in outer kinetochore function. J Cell Biol *155*, 1137-1145.
